# Construction of full-length Japanese reference panel of class I HLA genes with single-molecule, real-time sequencing

**DOI:** 10.1038/s41397-017-0010-4

**Published:** 2018-01-19

**Authors:** Takahiro Mimori, Jun Yasuda, Yoko Kuroki, Tomoko F. Shibata, Fumiki Katsuoka, Sakae Saito, Naoki Nariai, Akira Ono, Naomi Nakai-Inagaki, Kazuharu Misawa, Keiko Tateno, Yosuke Kawai, Nobuo Fuse, Atsushi Hozawa, Shinichi Kuriyama, Junichi Sugawara, Naoko Minegishi, Kichiya Suzuki, Kengo Kinoshita, Masao Nagasaki, Masayuki Yamamoto

**Affiliations:** 1 Tohoku Medical Megabank Organization, Tohoku University, Sendai, Japan; 2 Graduate School of Medicine, Tohoku University, Sendai, Japan; 3 National Research Institute for Child Health and Development, Tokyo, Japan; 4 Department of Pediatrics and Rady Children’s Hospital, University of California, San Diego, USA; 5 RIKEN Quantitative Biology Center, Osaka, Japan; 6 Graduate School of Information Sciences, Tohoku University, Sendai, Japan; 7 Tohoku University Hospital, Tohoku University, Sendai, Japan; 8 International Research Institute of Disaster Science, Tohoku University, Sendai, Japan; 9 Present Address: Department of Human Genetics, Graduate School of Medicine, The University of Tokyo, Tokyo, Japan

**Keywords:** Sequencing, Genome assembly algorithms

## Abstract

Human leukocyte antigen (HLA) is a gene complex known for its exceptional diversity across populations, importance in organ and blood stem cell transplantation, and associations of specific alleles with various diseases. We constructed a Japanese reference panel of class I HLA genes (ToMMo HLA panel), comprising a distinct set of HLA-A, HLA-B, HLA-C, and HLA-H alleles, by single-molecule, real-time (SMRT) sequencing of 208 individuals included in the 1070 whole-genome Japanese reference panel (1KJPN). For high-quality allele reconstruction, we developed a novel pipeline, Primer-Separation Assembly and Refinement Pipeline (PSARP), in which the SMRT sequencing and additional short-read data were used. The panel consisted of 139 alleles, which were all extended from known IPD-IMGT/HLA sequences, contained 40 with novel variants, and captured more than 96.5% of allelic diversity in 1KJPN. These newly available sequences would be important resources for research and clinical applications including high-resolution HLA typing, genetic association studies, and analyzes of cis-regulatory elements.

## Introduction

The human leukocyte antigen (HLA) system is the most variable gene complex in the human genome, and it encodes the major histocompatibility complex (MHC) proteins in humans. Accurate typing of HLA genes is particularly important in organ and blood stem cell transplantation where the risk of graft-versus-host diseases is known to increase with mismatching of HLA genes [1]. In addition, many gene-disease associations have been reported [2] between specific HLA alleles and susceptibility to autoimmune diseases [3] including rheumatoid arthritis [4], celiac disease [5], and type1 diabetes [6], to infectious diseases such as HIV [7, 8] and hepatitis C [9], and to other types of diseases such as narcolepsy [10].

Reflecting the extreme diversity of HLA genes, a growing number of HLA subtypes are being added to the IPD-IMGT/HLA database [11], in which the number of alleles is over 10,000. The allele and haplotype frequencies of HLA alleles are highly dependent on geographic location [12], which have been extensively used for analyzes of human migration and admixture history [13, 14]. Collection of population-specific HLA alleles is also important for accurate HLA typing [15, 16], imputation of HLA alleles [17], and microarray probe design [18].

Historically, a range of HLA typing methods have been developed from serological typing to nucleotide sequence typing. After the emergence of polymerase chain reaction (PCR) technology, methods for the locus specific amplification of DNA segments, typically, exons 2 and 3 in class I and exon 2 in class II HLA genes, followed by quantification with sequence-specific oligonucleotide probes (SSOPs) or sequencing based typing have been established [19]. The development of next generation sequencing (NGS) technologies, including pyrosequencing with emulsion PCR [20] and sequencing by synthesis technology with bridging PCR [21], have facilitated more accurate HLA typing with the information of other exons or entire HLA gene loci [22, 23]. However, reconstruction of novel haplotypes with short reads from NGSs is hampered by the limitation in the read length and, high variation due to insertions and deletions in the HLA regions of the reference genome. Recently, the advent of long read sequencing with single-molecule, real-time (SMRT) sequencing technology [24] has enabled assembling entire HLA gene sequences directly [25, 26].

As previously described [16, 27], we have identified the HLA class I alleles of 1070 Japanese samples recruited in a cohort project of the Tohoku Medical Megabank Organization (ToMMo), in which several alleles were not determined to full resolution (8-digit HLA typing). In this study, we focused on identifying full-length sequences of HLA-A, B, and C alleles for 208 samples that were not fully genotyped in the earlier analysis. To this aim, we designed sets of PCR primers for these HLA genes that consistently amplified all 208 samples. Using SMRT sequencing technology, we succeeded in reconstruction of full-length HLA-A, HLA-B, and HLA-C alleles and co-amplified HLA-H alleles. A distinct set of these HLA alleles was released as a ToMMo HLA panel, in which all the alleles covered the full-length of corresponding sequences in IPD-IMGT/HLA database. In addition, several regulatory sequence regions were newly identified and hundreds of variations were discovered in the extended regions. By comparing sequence differences between the panel and the database, we also identified dozens of novel alleles including nonsynonymous variants. Throughout the construction of the ToMMo HLA panel, we faced several technical challenges: full-length reconstruction of HLA alleles between designed primers, filtering out excess contigs, and assessment and correction of variants found in the alleles. We overcame these challenges with a newly devised, robust pipeline named Primer Separation Assembly and Refinement Pipeline (PSARP), which successfully reconstructed a set of full-length and quality enhanced alleles of HLA class I genes from SMRT sequencing and short-read data.

## Materials and methods

### Sample selection

This study was performed as part of the prospective cohort study at the Tohoku Medical Megabank Organization (ToMMo) with the approval of the ethical committee of the Tohoku University School of Medicine. All cohort participants are residents of Miyagi Prefecture, Japan, and provided their written consent. Whole genome sequencing (WGS) was performed for 1070 cohort participants (1KJPN) [27], where short-read data (162 bp paired-end, 32.4x on average) were generated on the Illumina HiSeq 2500 (Illumina, San Diego, CA, USA). Genotypes of HLA-A, HLA-B, and HLA-C in 1KJPN were inferred from the WGS data with HLA-VBseq [16], in which not all of the genotypes were determined within IPD-IMGT/HLA subtypes for 220 individuals. All these samples were selected for the subsequent analyzes. DNA extraction methods used have been described previously [27].

### Primer design and PCR

PCR primer sets were designed for amplifying HLA-A, HLA-B, and HLA-C alleles to cover full-length genomic sequences deposited in the IPD-IMGT/HLA database [11]. The corresponding coordinates of target regions on hg19 reference genome and the primer sequences are described in Supplementary Table 1. A manual inspection of the primer annealing sites for each sample was performed using the WGS data mapped on hg19 reference genome with BWA-MEM and Integrative Genomics Viewer (IGV) [28]. Searching for other possible annealing sites for the primer sets in the hg19 reference genome by was performed with BLASTN version 2.2.27+ with a maximum *E* value of 0.1. The sites with an edit distance <2 are listed in Supplementary Table 2. The index sequences were designed following the guidelines listed the shared protocols of Pacific Biosciences (Menlo Park, CA, USA) (Supplementary Table 3). The 12 index sequences (16 bp) with the universal sequences (5 bp) were ligated to the 5′ ends of the primers in preparation for multiplex sequencing.

A PCR reaction was performed with each DNA sample using each of the primer sets separately. Each 30 μl PCR mixture was prepared with 0.75 U PrimeSTAR GXL DNA Polymerase (Takara, Tokyo, Japan), 44 ng template DNA, 0.2 μM each of forward and reverse primer, and 0.2 mM dNTP in 1x PrimeSTAR GXL buffer. Cycle condition was 25 cycles of 10 s at 98 °C, 15 s at 60 °C, and 6 min at 68 °C. PCR products amplified with the A, B, and C primer sets from each sample were pooled.

### SMRT sequencing of PCR products

Libraries for sequencing were constructed from pooled DNA for each sample with a DNA Template Prep Kit 2.0 (3–10 kb) (Pacific Biosciences) following the supplier’s instructions. The 220 indexed libraries were sequenced with a PacBio RS II instrument (Pacific Biosciences) across 19 cells, using P6-C4 chemistry for 24 libraries in two cells for 4 h movie time and P4-C2 chemistry for 196 libraries in 16 cells for 3 h movie time. Each cell contained 8–12 libraries with library-specific barcode sequences and sequence data were separated in downstream analyzes.

### Initial analysis of PCR products

SMRT sequencing reads from each run were assembled and split into sample-specific contigs by AmpliconAnalysis software, which was included in SMRT analysis version 2.3.0, using the default parameters. The assembled contigs were mapped with BWA-MEM to genomic sequences registered in the IPD-IMGT/HLA database. The HLA gene name of each contig was determined to that of the corresponding database subtype, which was the sequence mapped by the longest fragment of the contig. To determine whether an assembled contig contained the sequences of the designed primers, all the A, B, and C primer sequences were mapped to the contig with BWA-MEM. The contig was considered to contain a primer sequence if the sequence was mapped to the contig with edit distance <2.

### Bioinformatics methods: PSARP

**Fig. 1:**
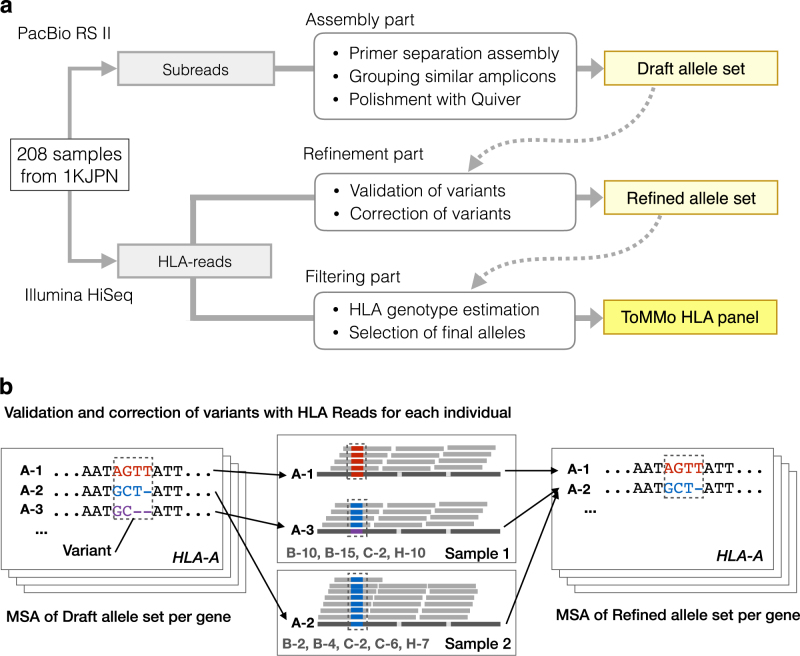
ToMMo HLA panel construction with PSARP. **a** Overview of ToMMo HLA panel construction. The workflow of constructing the ToMMo HLA panel by primer separation assembly and refinement pipeline (PSARP). PSARP consists of three subparts: assembly, refinement, and filtering. The details of each part are described in the Materials and Methods. **b** Illustration of refinement in PSARP. The left panel of the figure shows a multiple sequence alignment (MSA) of *HLA-A* gene in the Draft HLA panel, which is used for variant identification. In the middle panel, the variant validation process is illustrated, in which Sample 1 has heterozygous draft alleles for *HLA-A* (A-1 and A-3) and Sample 2 has homozygous alleles (A-2). For a variant in sample 1, “AGTT” in A-1 is supported by WGS reads, whereas “GC--” in A-3 is not supported, as WGS read was “GCT-”. For Sample 2, the variant for A-2 allele is supported. After the correction process, A-3 is merged into A-2. The right panel shows the Refined HLA panel derived from the correction process

PSARP consisted of three parts: (a) Assembly of alleles that had whole gene lengths between primer sequences from SMRT-sequencing data, (b) Refinement of the assembled alleles, and (c) Filtering of low confidence alleles (Fig. 1a). In Assembly part, all the SMRT subreads were assembled and processed into draft alleles for each sample. A set of the assembled alleles in this part was denoted the Draft allele set. For refinement, variations in the Draft allele set were identified by multiple sequence alignment (MSA) [29]. Then, Draft alleles were validated and refined for each individual allele at the variant positions (Fig. 1b) using short-read sequencing data mapped surrounding HLA genes (HLA-reads). The resultant alleles were denoted the Refined allele set. For filtering, HLA genotypes of each individual allele were estimated from the HLA-reads within the Refined allele set and IPD-IMGT/HLA database. Finally, the ToMMo HLA panel was selected from the Refined allele set by filtering less confident alleles in the set. The details of PSARP are described in Supplementary Methods and Supplementary Figure 1.

### Code availability

The source code for PSARP is available at http://nagasakilab.csml.org/data/psarp_scripts.zip

## Results

### Amplicon sequencing and initial genome assembly

**Fig. 2:**
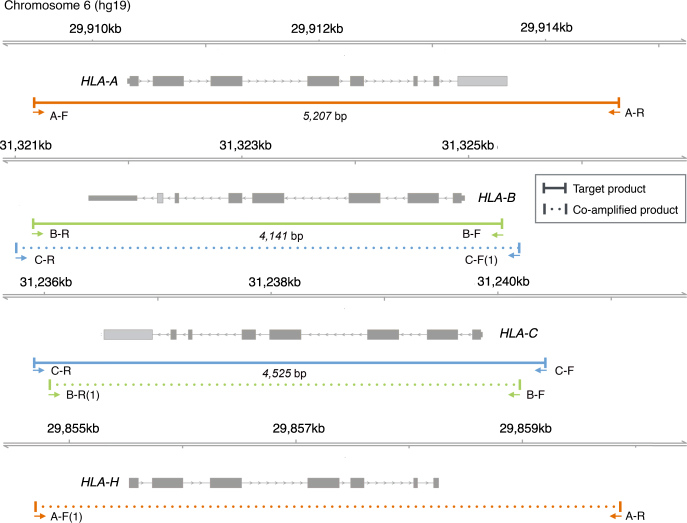
Overview of PCR products of designed primers. Locations of 5′-end and 3′-end primer sequences for *HLA-A, HLA-B*, and *HLA-C* genes are shown in hg19 coordinated with the target and co-amplified PCR products. Primer names “A–F” and “A–R” stand respectively for forward and reverse primers for *HLA-A* gene. For each of “A–F(1)”, “C–F(1)”, and “B–R(1)”, the edit distance of the primer sequence from the corresponding sequence in hg19 reference is shown in parenthesis after the primer name

We designed sets of PCR primers to amplify the full-lengths of HLA-A, HLA-B, and HLA-C alleles from the 220 samples selected from 1KJPN. The sets of primer sequences were designed from conserved regions in all 220 samples; the conservation was confirmed by manual inspection. Locations of the designed regions on hg19 reference genome are summarized in Fig. 2. We confirmed that all the PCR products from the 220 samples were successfully amplified for A, B, and C primer sets, and were the same size as that for a positive control sample, as shown in Supplementary Figure 2. These products were subjected to SMRT sequencing. The average number of polymerase reads per sample and the average polymerase read length were 7411 and 13,385, respectively, for P6-C4 runs, whereas the average reads were 5247 and 5035 for P4-C2.

After the assembly of the SMRT sequencing data for the 220 samples, all the contigs were classified into four HLA types: A, B, C, and H, which were expected from potential primer annealing sites on the hg19 reference genome (Fig. 2). Although the 220 samples were randomly assigned into 21 SMRT cells, 8 of 12 samples in a cell had contigs that classified into the same HLA-B and HLA-C subtypes. As the cell was potentially contaminated, we excluded the 12 samples in the cell and remained 208 samples for the subsequent analyzes. The number of alleles in a sample for each gene was assumed to be one or two, corresponding to its homo/heterozygosity. However, only 31, 3, and 7 of the 208 samples had one or two contigs of HLA-A, HLA-B, and HLA-C, respectively. The majority of the samples had more contigs, as many as 8 (Supplementary Table 4). Therefore, a reduction of contigs was necessary for determining a reliable set of alleles. For HLA-H, alleles of 24 samples were estimated as homozygous deletions from copy number analysis at the locus using the WGS data (Supplementary Methods). Among the remaining 184 samples, 160 were successful in assembling HLA-H contigs (Supplementary Table 4). The miss of contigs in the 24 samples were possibly accounted for by a failure in primer hybridization as our primers were designed to capture HLA-A, HLA-B, and HLA-C alleles.

The assembled contigs were examined if they were full-length i.e., covering the entire region between designed primers. While the rate of full-length contigs for each HLA-A, HLA-B, and HLA-H was over 90%, that of HLA-C was as low as 29% (Supplementary Table 5). The failure in full-length reconstruction generally occurred for HLA-C alleles that were also amplified with the B primer set. Since the potential annealing sites for the B primer set were located in the inner region of those for the C primer set (Fig. 2), the number of sequencing reads was enriched in the inner region compared to the outside regions. We hypothesize that this difference led to truncation of the outside regions during the assembly process.

### Construction of ToMMo HLA panel by PSARP

To overcome two challenges encountered in the preliminary evaluation, we developed the Assembly part of PSARP (Fig. 1a) and applied it to the SMRT sequencing data. One of the challenges was reconstructing whole length of alleles between the designed primer set, particularly for HLA-C. This was resolved with a separation of the sequencing reads by associated primer sets before Amplicon Analysis (Supplementary Methods). With this processing, all the samples were successful in obtaining full-length HLA-C alleles. The other challenge was a reduction of excess contigs. This was addressed through the processes from “Grouping and fusing contigs” to “Post processing” (Supplementary Figure 1a), in which similar contigs were merged and ones with low support reads were discarded.

Thus, we succeeded in obtaining possible numbers of full-length alleles, which was 1 or 2, for each of HLA-A, HLA-B, and HLA-C genes in all the 208 samples (Supplementary Table 6). The number of HLA-H alleles per sample was 0–2, which was also consistent with the existence of deletion polymorphisms at the locus. The number of unique alleles identified from the Assembly was 336 in total, which we called the Draft allele set.

Evaluation and refinement of sequence quality in the Draft allele set were addressed by refinement in PSARP (Fig. 1a). In the evaluation, variant of alleles was defined as sequence difference among alleles for each gene (Fig. 1b). The numbers of variant positions in the Draft allele set were 306, 223, 231, and 131 for HLA-A, HLA-B, HLA-C, and HLA-H, respectively. Variants in each allele were examined with the HLA-reads (Fig. 2) from samples that had the allele as their draft. As a result, the mean variant accuracy was 97.3% in the Draft allele set (Supplementary Table 7), in which 73,855 variants were examined in total. Details of the examination were described in Supplementary Methods.

With a variant correction process (Fig. 1b), 33, 168, 22, and 103 variants were corrected in HLA-A, HLA-B, HLA-C, and HLA-H alleles, respectively. Subsequently, 248 unique alleles were obtained and named the refined allele set. The number of unique alleles in the refined allele set was significantly reduced from that in the draft allele set, despite of the small amount of variant correction, which was only 0.44% of the examined variants. The variant accuracy in the refined allele set was increased from that of the draft allele set for all the genes, in which the mean accuracy was 98.0% (Supplementary Table 7).

Through the filtering part of PSARP, a more reliable set of alleles were selected from the refined allele set. Specifically, the alleles that recursively genotyped with HLA-VBSeq were selected (Supplementary Methods). In this filtering process, several mistyped alleles were altered with more suitable ones. Examples of these alternations are illustrated in Supplementary Figure 3, in which WGS reads mapped on a selected allele of a sample were extracted and mapped on a discarded allele of the sample for comparing mapping status around differed positions between the two alleles. Finally, the resultant set of 139 distinct alleles was cataloged as the ToMMo HLA panel (Supplementary Data 1a). The mean variant accuracy of the panel reached 99.1%, which was significantly improved from that in the Refined allele set (Supplementary Data 1b). The numbers of alleles and the variant accuracies throughout the panel construction are summarized in Supplementary Table 4.

### Reference extension and variant discovery

As expected from our primer design, all the 139 alleles found in the ToMMo HLA panel had longer sequences compared with the closest ones in the IPD-IMGT/HLA database. Particularly, large numbers of regulatory regions, which were upstream and downstream of coding regions, were newly covered in the ToMMo HLA panel (Supplementary Figure 4, 5). The mean length of the alleles and their regulatory regions for each gene are shown in Table 1, in which corresponding values of the compared database alleles are shown in parentheses. Additionally, the regulatory regions contained 192 variant positions that were newly identified in the panel. Density of variants in the identified alleles was examined for these regulatory regions, exons, and introns for each gene (Supplementary Methods, Supplementary Figure 6). Generally, the density in the regulatory regions was comparable to that in the exons and introns, except for enrichment or depletion of the density in some exons and introns for HLA-A, HLA-B, and HLA-C.

**Table 1:** Summary of sequence extensions in the ToMMo HLA panel

		HLA type				
		A	B	C	H	Total
Number of alleles		26	67	37	9	139
Mean length		5171 (3470)	4098 (3239)	4465 (3350)	5155 (3457)	–
Upstream region	Mean length	833 (284)	338 (250)	546 (282)	820 (299)	–
	Novel variants	27	2	9	9	47
Downstream region	Mean length	1425 (273)	1077 (307)	1022 (171)	1437 (261)	–
	Novel variants	46	37	35	27	145

*HLA* human leukocyte antigen, *ToMMo* Tohoku Medical Megabank Organization

### Characteristics of novel discovered alleles

To characterize the differences between newly identified alleles and the known ones, every allele in the ToMMo HLA panel was compared with the closest ones in the IPD-IMGT/HLA database by pair-wise alignment (Supplementary Methods). All the compared alleles are shown in Supplementary Figure 4 and 5 with their edit distances within exons and noncoding regions. In total, 40, 12, and 10 alleles were novel up to 8-digit, 6-digit, and 4-digit resolution, which corresponded to having genomic, exonic, and nonsynonymous variants from the known subtypes, respectively (Table 2). There were 8 alleles whose intron regions were newly identified because of the known subtypes in the database had been only cataloged with cDNA sequence. In the three coding genes (HLA-A, HLA-B, and HLA-C), 7 alleles had novel exonic variants to the closest database subtypes (Table 3). While most of the alleles had 1 or 2 substitutions to the closest known subtypes, B_00063 had up to 7 substitutions to B*56:04. In contrast to the three coding genes, a greater ratio of HLA-H alleles (5 of 9) had novel exonic variants. The difference in ratio might reflect that HLA-H is a pseudogene and is much less cataloged in the database compared with the three coding genes.

**Table 2:** Novel alleles in ToMMo HLA panel compared with the closest subtypes in IPD-IMGT/HLA database

	HLA type				
	A	B	C	H	Total
Novel up to 8-digit	8	21	5	6	40
Add intron sequences	1	3	1	3	8
Novel up to 6-digit	2	4	1	5	12
Novel up to 4-digit	2	2	1	5	10

*IPD* immuno polymorphism database, *IMGT* international ImMunoGeneTics information system, *HLA* human leukocyte antigen, *ToMMo* Tohoku Medical Megabank Organization

**Table 3:** Variants in coding region of novel alleles

ToMMo HLA	Freq.	IMGT/HLA	Exon	Pos.	Var.	AA pos.	AA alt.
A_00012	1	A*11:01:01	2	53	A > G	43	K > E
		A*11:77	4	256	A > G	292	K > E
A_00021	1	A*26:03:01	3	73	A > G	139	Q > R
B_00021	2	B*39:02:01	2	173	A > G	Synonymous	
		B*39:02:02	5	113	T > C	Synonymous	
B_00028	1	B*40:01:02	3	254	G > C	Synonymous	
B_00053	1	B*54:01:01	2	69	G > T	48	A > S
B_00063	2	B*56:04	3	20	G > C	121	R > S
			3	76	TA > AC	140	L > Y
			3	120	A > C	155	S > R
			3	134	G > C	Synonymous	
			3	216	CT > AC	187	L > T
C_00014	1	C*03:04:01	3	63	G > A	136	G > R

*IMGT* international ImMunoGeneTics information system, *HLA* human leukocyte antigen, *ToMMo* Tohoku Medical Megabank Organization, *Freq.* frequency, *Pos.* position, *Var.* variant, *AA* amino acid, *alt.* alternative

### HLA allele distribution of the 208 samples

**Fig. 3:**
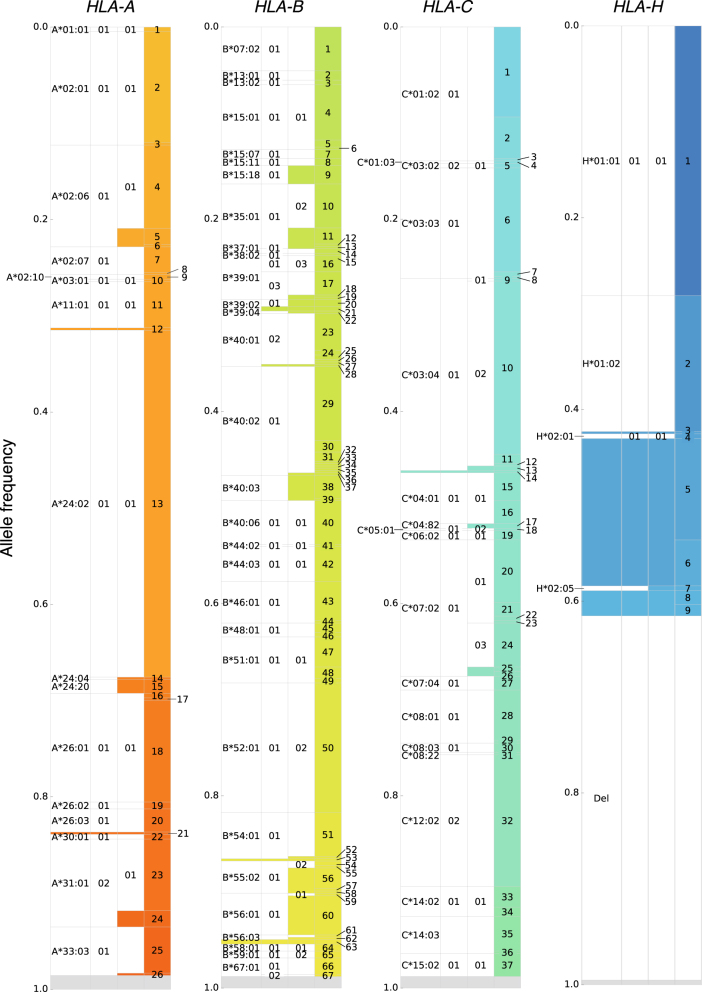
Allele distribution of 208 samples in the ToMMo HLA panel. The alleles in the ToMMo HLA panel are shown for each HLA gene, in which each row corresponds to a unique allele and its width is proportional to the allele frequency within the 208 samples. Four inner columns of each row correspond to the 4-digit, 6-digit, and 8-digit IMGT/HLA names of the allele and the allele itself from left to right. A rectangle filled with color indicates a novel sequence identified in the panel, except that a gray color fills untyped alleles. The rightmost column is fully filled with colors since every allele in the panel has novel external sequence that is not found in the database

Genotypes of HLA-A, HLA-B, HLA-C, and HLA-H for the 208 samples in the ToMMo HLA panel were determined with HLA-VBSeq using the WGS data, in which copy numbers of HLA-H locus were identified in advance and considered in the genotype estimation (Supplementary Methods). Among the 416 alleles of 208 samples for each gene, 98.9% were typed in average. Allele distribution of each gene in the 208 samples is shown in Fig. 3. In summary, the total frequencies of alleles up to 8-digit resolution in the 208 samples were 6.0, 17.1, 2.1, and 18.8% for HLA-A, HLA-B, HLA-C, and HLA-H, respectively.

### Coverage of HLA alleles in the 1KJPN population

**Fig. 4:**
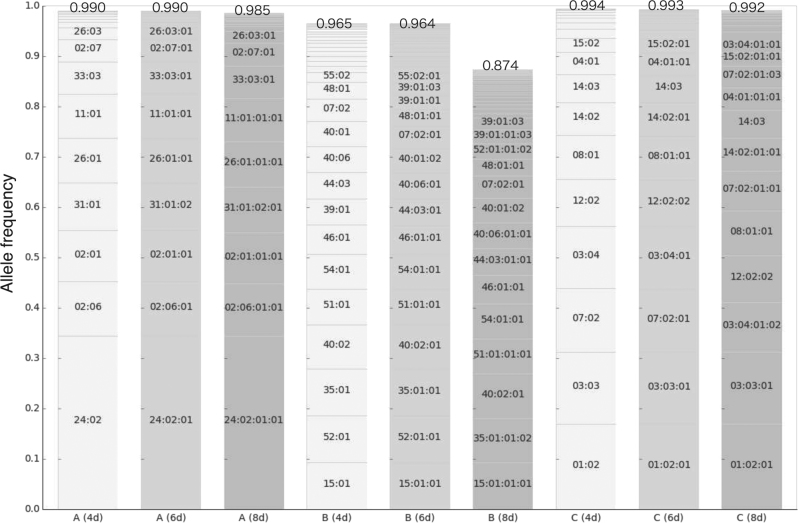
Coverage of 1KJPN allele distribution within the ToMMo HLA panel. A distribution of 1KJPN alleles that were covered within the ToMMo HLA panel for each combination of *HLA-A, HLA-B*, and *HLA-C* genes at 4-digit, 6-digit, and 8-digit resolutions is shown. The figures on top of the stacked bars are overall fractions of 1KJPN alleles that were covered within the panel. Allele names are shown for those with frequencies >2% in the 1KJPN population

The coverage of allele distribution in the 1KJPN population [27] with the ToMMo HLA panel was estimated for HLA-A, HLA-B, and HLA-C genes. In the estimation, HLA genotypes for the 208 samples were determined within the panel. The genotypes of the least of samples from 1KJPN were estimated with HLA-VBSeq using IPD-IMGT/HLA release 3.24 database. The coverage of 1KJPN allele distribution by the panel was 99.0, 96.5, and 99.4% at 4-digit resolution, for HLA-A, HLA-B, and HLA-C, respectively. The similar coverages at 6-digit and 8-digit alleles are shown in Fig. 4. Although the 208 samples for the panel had been selected from those with untyped alleles within the database, the ToMMo HLA panel covered a significant number of alleles existing in the 1KJPN population.

## Discussion

We have exploited SMRT-sequencing technology to develop a collection of alleles for HLA class I genes, namely HLA-A, HLA-B, HLA-C, and HLA-H, from 208 samples from a Japanese cohort. PCR primers for HLA-A, HLA-B, and HLA-C were designed to cover the entire region of current alleles registered in the IPD-IMGT/HLA database, in which HLA-H alleles were additionally obtained with the HLA-A primer set. During a construction of a reliable HLA allele set from these assembled data, we encountered several problems. First, the number of assembled alleles for each gene of an individual frequently exceeded 2, in which multiple alleles were assembled even for single PCR products. Second, a large fraction of HLA-C alleles was not assembled with their expected lengths. Finally, since we sequenced hundreds of samples, we found several mistyped variants, although the mistyping rate was low. Therefore, validation and correction of sequence differences were essential to reduce redundancy and enhance quality of the allele set. These three problems were overcome with the newly devised PSARP, in which short-read sequencing data from the same samples were used for quality assessment, enhancement, and selection of the sequences. Although several methods have been proposed for *de novo* assembly of SMRT sequencing data [30, 31] and additional use of short-read data [32, 33], no single approaches exist for constructing a locus-specific reference panel from hundreds of SMRT sequencing data in such a systematic way.

With PSARP, we succeeded in constructing a set of 139 HLA alleles from the 208 samples, which we named the ToMMo HLA panel. We have investigated several contributions of this panel to the current collection of class-I HLA alleles registered in the IPD-IMGT/HLA database [11]. Our primer design enabled identification of novel regulatory regions for all the alleles. The panel contained 192 novel variations in the regulatory regions, which could potentially affect regulation of gene expression [34]. The number of discovered alleles with genomic variants reached 40. Among them, introns of 8 alleles were newly identified in the panel, and 7 were novel coding alleles with exonic variants. The number of identified HLA-B alleles was 67, which was the highest among the 4 genes. This result was consistent with previous studies [11, 35], which showed HLA-B was the most divergent gene among class-I genes. The total frequency of novel alleles in the 208 samples for HLA-B was 17.1%, which was also the highest among the genes in the panel. For HLA-H, which had only 12 known alleles and was less frequent in the database, 6 of the 9 alleles in the panel had novel genomic variants. As HLA genes are in high levels of linkage disequilibrium, microarray typing of novel variants found in both the three coding genes and HLA-H would potentially enhance genotype imputation quality in the HLA super locus.

All the sequences identified in the ToMMo HLA panel are available (Supplementary Data 1a). We expect that the panel will be particularly useful in genotype imputation as there have been number of studies evaluating that inclusion of ethnic specific reference panel improves an imputation accuracy in both whole genome [36–38] and HLA loci [39, 40]. In addition, full genomic sequences including introns and regulatory regions are determined in the panel and are highly informative in HLA typing from WGS data [16, 41]. The panel can be also expected to be used for novel microarray probe design [18, 36] and identification and analyzes of cis-regulatory elements [8, 42]. The panel is especially suitable for the Japanese population as the coverage of 1KJPN allele distribution in 4-digit resolution is 99.0, 96.5, and 99.4% for HLA-A, HLA-B, and HLA-C alleles, respectively. Nevertheless, the additional collection of class-I alleles is also valuable to cover rare haplotypes and variations at higher resolution, especially for HLA-B.

In this study, we have focused on sequencing of class I HLA genes. Recently, GenDX have offered commercial primers for full-length sequencing of HLA-A, -B, and -C genes [43] using SMRT sequencing, in which the mean length is up to 3500 bp. Our primers are available in public domain, can cover broader regions including regulatory sequences (Table 1), and have achieved collection of HLA-A, -B, -C and -H alleles from 208 Japanese samples with the aid of PSARP. An important future direction is a construction of full-length reference panel of class II HLA genes, which are also known to be highly varied and clinically important. GenDX designed primers to amplify exon 2 to 4 of HLA-DRB1 and -DQB1 genes, in which the mean length of their consensus sequences is up to 4000 bp [43]. Although full-length reconstruction of class II HLA genes, whose lengths are typically over 6000 bp, seems more challenging, it would be overcome by advance in sequencing chemistry or other long read technology such as Oxford Nanopore sequencing [44]. The strategies of PSARP would also be applicable to analyze class II HLA genes and other complex regions that consist of multiple genes such as KIR gene cluster, and loci with copy number variations [45].

### Data availability

DNA sequences of ToMMo HLA panel are available from DDBJ under accession numbers LC326108 to LC326246. Sequence data will be available on request after approval of the Ethical Committee and the Materials and Information Distribution Review Committee of Tohoku Medical Megabank Project. Part of the data is available as open data from the National Bioscience Database Center website under the accession hum0015.

## Electronic supplementary material

Supplementary Information

Supplementary Table 2

Supplementary Table 3

Supplementary Data 1a

Supplementary Data 1b
