# Supplementary material for: Construction of full-length Japanese reference panel of class I HLA genes with single-molecule, real-time sequencing
Source: Pharmacogenomics J. 2018 Jan 19;19(2):136–46. doi: 10.1038/s41397-017-0010-4 (PMC6462828; doi:10.1038/s41397-017-0010-4)
Supplement: Supplementary file 1 — Supplementary Information [file 41397_2017_10_MOESM1_ESM.pdf]

Supplementary information for the paper "Construction of full-length Japanese reference panel of class I HLA genes with single-molecule, real-time sequencing"

This document is a companion to the paper "Construction of full-length Japanese reference panel of class I HLA genes with single-molecule, real-time sequencing". It contains Supplementary Methods, Supplementary Figures, and Supplementary Tables.

Supplementary Methods **1**
Bioinformatics methods: PSARP 1
Copy number analysis of HLA genes 4
HLA genotype estimation with HLA-VBSeq 4
Classification of HLA alleles 4
Annotations for HLA alleles 5
Variant density analysis 5
Evaluation of variant accuracy 6
Supplementary References **6**
Supplementary Figures **8**
Supplementary Tables **13**

Supplementary Methods

Bioinformatics methods: PSARP

Overview

PSARP consisted of three parts: (a) Assembly of alleles that had whole gene lengths between primer sequences from SMRT-sequencing data, (b) Refinement of the assembled alleles, and (c) Filtering of low confidence alleles (Figure 1a). In Assembly part, all the SMRT subreads were assembled and processed into draft alleles for each sample. A set of the assembled alleles in this part was denoted the Draft allele set. For refinement, variations in the Draft allele set were identified by multiple sequence alignment (MSA). Then, Draft alleles were validated and refined for each individual allele at the variant positions (Figure 1b) using short-read sequencing data mapped surrounding HLA genes (HLA-reads). The resultant alleles were denoted the Refined allele set. For filtering, HLA genotypes

of each individual allele were estimated from the HLA-reads within the Refined allele set and IPD-IMGT/HLA database. Finally, the ToMMo HLA panel was selected from the Refined allele set by filtering less confident alleles in the set.

Assembly part of PSARP: Construction of Draft allele set

Schematic workflow of Assembly is shown in Supplementary Figure 1a. First, SMRT sequencing reads of each sample, which we refer to as “Sample Subreads”, were extracted from subreads that were sequenced in the same run of PacBio RS II. Then, Sample Subreads whose length was over 300 bp were assembled into contigs (Sample Contigs) by AmpliconAnalysis software included in SMRT analysis version 2.3.0 with default parameters, except that maxReads and maxPhasingReads were both set to 20,000. Additionally, primer-specific (PS) subreads, that were likely to contain a primer sequence, were extracted from Sample Subreads for each of A and C primer sets. Specifically, a subread that contained substrings matched to either one of a primer pair in less than five mismatch bases was regarded as a PS subread. Then, PS Subreads were assembled into “PS Contigs” by the same procedure for Sample Contigs, for each of the primer sets. As Sample Contigs and PS Contigs could contain redundancy in the same PCR product, all these contigs were grouped and fused into “Candidate alleles” using the following steps:

1. For every pair of the contigs, generate a pairwise sequence alignment with BLASTN program[1].
2. Classify each pair of contigs into the same group if the numbers of mismatches between them is ≤ 10 and overhangs at the ends are ≤ 50 bp.
3. For every group of contigs, generate a multiple sequence alignment (MSA) of the contigs with Clustal Omega program[2].
4. Generate a candidate allele from the MSA for each group, in which bases of the allele are determined by taking majority base at every alignment position.

These candidate alleles were iteratively updated by mapping Sample Subreads on the candidate alleles with pbalign, then polishing the alleles with Quiver (both programs were included in SMRT Analysis version 2.3.0)[3]. This process was iterated until no bases were updated or the number of iterations exceeded the maximum number, which was set to seven.

After the polishing with Quiver, Candidate alleles were processed into Draft alleles by the following steps, which corresponded to “Post processing” in Supplementary Figure 1:

1. Find a roughly matched HLA subtype (e.g. A*01:01:01:01) for each allele by mapping the allele sequence to all of the genomic sequences registered in the IPD-IMGT/HLA database[4] with BWA-MEM[5]. A database sequence with the longest alignment length is regarded as the matched subtype.
2. Remove primer sequences from each end of Candidate alleles, in which the primer regions for each allele are detected by mapping a set of primer sequences for the gene determined in Step 1.
3. For each allele, adjust sequence strand to the same direction as that of a matched subtype.

4. Discard Candidate alleles that are still modified with the last Quiver process. Then, the allele sequences are ranked with an amount of Sample Subreads mapped on the alleles by pbalign. For each gene, the top two contigs are retained, except when the second best contig has less than 25% of the total subreads for the same gene, then only the best contig is retained.

Finally, Draft alleles for the 208 samples were merged into Draft allele set, in which the same alleles were listed only once.

Refinement part in PSARP

The Refinement by PSARP is shown in Supplementary Figure 1b. For each sample, HLA-reads that were paired-end short-read WGS data mapped around HLA genes were extracted. Specifically, the HLA-reads contained the pair of reads, in which both reads were unmapped, or either of the pair was mapped on regions of *HLA-A, -B, -C, -DM, -DO, -DP, -DQ, -DR, -E, -F, -G, -H, -J, -K, -L, -N, -P, -S, -T, -U, -V, -W, -Z, -AS1*, or their 5 kb flanking regions. These gene regions were annotated in Ensembl[6] (release 75). The HLA-reads for each sample were mapped on the Draft alleles of the same sample with BWA-MEM, which were referred as “Mapped HLA-reads”. The variant list, which was a collection of variants identified in the Draft allele set, was identified from a MSA of the alleles in the Draft allele set for each gene (The left panel of Figure 1b). These MSAs were created using MAFFT v7.058b[7, 8] with an option “--maxiterate 10”. Using the Mapped HLA-reads and the variant list in the Draft allele set, Draft alleles were examined and corrected if needed for every variant position for each sample, which is illustrated in the middle panel of Figure 1b. Specifically, a variant in a draft allele was considered to be “valid” if at least two support-reads exist, where a support-read of a variant was an HLA-read that contained an exact region of 20 bp length started from 3 bases upstream of the variant. Note that the reads with more than 4 edit distance to the sequence were excluded in advance. If the variant was considered to be invalid according to the former condition, the other variants observed at the same position were set as candidates for an alternative variant (The left panel of Figure 1b). Then, the invalid variant was replaced with one of the candidates (ALT) if the sum of support-read number for the variants other than ALT was less than half of that for ALT.

Filtering part in PSARP: Construction of the ToMMo HLA panel

Filtering by PSARP is shown in Supplementary Figure 1c. A custom reference for HLA genotype estimation was created by combining genomic sequences in the IPD-IMGT/HLA (release 3.24) database and Refined allele set, in which the database sequence that was completely covered by any of sequences in the panel with no mismatches was excluded. Then, HLA genotypes for each sample, which were selected alleles from the custom HLA reference, were estimated with HLA-VBSeq[9]. The details of the genotype estimation are described in the subsequent sections, in which copy number variants found at the HLA-H locus was also considered. Using the estimated genotypes, an allele in the Refined allele set was removed if there were no samples that had the allele as both the genotyped

one and assembled one. Alleles that were assembled as only homozygous were also removed. We designated the remaining set of alleles as the ToMMo HLA panel.

Copy number analysis of HLA genes

For the copy number analysis of HLA genes, whole genome sequencing (WGS) data from 1,070 samples in the 1KJPN study[10] was used. The sequencing data was aligned to the hg19 human reference genome with bowtie2 aligner, followed by copy number analysis using GenomeSTRiP2 [11] with following parameters: tilingWindowSize, 1000; tilingOverlap, 500; maximumReferenceGapLength, 1000; boundaryPrecision, 100; minumumRefinedLength, 500. From the results, copy number calls overlapped with HLA-A, -B, -C, and -H genes were extracted for the 208 samples that were used for building ToMMo HLA. In this study, no copy number variations were detected for HLA-A, -B and -C. For HLA-H, 74, 110, and 24 samples had 2, 1, and 0 copies of the gene, respectively.

HLA genotype estimation with HLA-VBSeq

For a determination of HLA genotypes from the HLA-reads, the most probable alleles were selected from HLA-VBSeq results according to the following procedures. First, the depth of coverage for each reference was calculated from Z values, which were proportional to the estimated abundance of WGS reads. The relative depth (RD) of each reference was calculated as the estimated depth of coverage divided by the sum of those for the same HLA gene. For genes with 2 copy numbers, the top 2 references as ranked by RD were retained, in which references with RD less than 0.15 were discarded from the candidates. When the 2 candidates remained, the genotype was determined to be homozygous (HOM) for the first allele if RD of the first allele was over 0.5 and was 2.5 times greater than that of the second one; otherwise, the genotype was called as heterozygous (HET) for the 2 alleles. When the remaining candidate had only 1 copy number, the genotype was HOM if RD was over 0.5, otherwise is was HET for the allele and an untyped allele. For genes with 1 copy number, the top reference was called if the RD was over 0.5, otherwise it was called untyped.

Classification of HLA alleles

Each allele in the ToMMo HLA panel was compared with known subtypes registered in the IPD-IMGT/HLA database (release 3.24) and classified with the nomenclature system for the database, in which 4-, 6-, and 8-digit names (e.g. A*01:01:01:01) discriminated differences in amino acids, exon sequences, and genomic sequences, respectively. For each ToMMo HLA allele, all the cDNA sequences whose gene name was the same as the allele's and that covered all the exons were selected from the database, then mapped to the allele by the est2genome program included in EMBOSS-6.5.7[12] with options "-intronpenalty 40 -splicepenalty 1 -mismatch 1 -align 1 -usesplice". From the mapping

result, the closest 6-digit names of the allele were determined to be those with the minimum edit distance (MED) from the allele among the cDNAs. We defined that if the MED was 0, the allele was known up to 6-digit. Translated amino acids for the allele and the closest 6-digit subtypes were also examined, and when they agreed, the allele was considered as known up to 4-digit, otherwise novel. On the other hand, genomic sequences whose gene name was the same as the allele's were extracted from the database and pair-wise sequence alignments (PSAs) were made between those and the allele with BLASTN program. Then, the closest 8-digit names were determined to those who had the 6-digit prefix contained in the closest 6-digit names and had the MED from the allele. If the MED was 0, the allele was known up to 8-digit.

Annotations for HLA alleles

For each allele sequence identified in ToMMo HLA panel, locations of exons and known sequences registered in IPD-IMGT/HLA database were annotated using the cDNA and gDNA sequences of their closest subtypes as follows. First, Each cDNA sequence was split aligned to the corresponding allele with est2genome (See "Classification of HLA alleles") and alignment gaps were filled with 'N' bases. Then, for each of HLA-A, -B, -C, and -H genes, MSA of alleles, their closest gDNAs (if existed) and the gap filled cDNAs was created using MAFFT with an option "--maxiterate 10". Note that we had to manually shift alignment positions of the last exon for several HLA-A cDNA alignments to meet with those presented in the database. There were multiple candidate positions of the exon on the allele because of a short length of the exon, which was only five bases: "TGTGA". From the MSAs, we presented layout of alleles, the closest known subtypes in the databases, and the exons for each of HLA-A, -B, -C, and -H genes in Supplementary Figure 4 and 5.

Variant density analysis

Variant density in the discovered HLA alleles was analysed for regulatory regions, introns, and exons for each of HLA-A, -B, -C, and -H genes. MSA of alleles and their closest sequences in the IPD-IMGT/HLA database for each gene was created in advance. The variant density of a region for alleles was defined as a proportion of bases that had variants within the alleles to the length of the region in the MSA. The 95% confidence interval of the estimated density was calculated based on Clopper-Pearson method. Coordinates of 5' upstream and 3' downstream regulatory region, exons, and introns were determined for those of TMM_HLA_A_00013, TMM_HLA_B_00029, TMM_HLA_C_00010, TMM_HLA_H_00001 for HLA-A, -B, -C, and -H genes, respectively. Results of the analysis for alleles in ToMMo HLA panel were shown in Supplementary Figure 6.

Evaluation of variant accuracy

To evaluate overall quality of the Draft allele set, the Refined allele set, and ToMMo HLA panel, MSAs of allele sequences for each of HLA-A, -B, -C, and -H genes in each panel were created. Variant sites were identified from each MSA as alignment columns that had multiple unique bases. Consecutive variant sites were regarded as a single variant site with multiple bases. The left panel of Figure 1b illustrates variant identification from MSA of HLA-A alleles in the Draft allele set. The number of variant sites identified in each gene of each panel was summarized in a column "Variant sites" of Supplementary Table 7.

Variants on each allele were examined with WGS data from samples that had the allele as an assembled one. We defined that a variant was supported if at least two support-reads existed in WGS data of a sample having the allele, where a support-read was an HLA-read that contained an exact region of 20 bp length started from 3 bases upstream of the variant. We estimated an accuracy of variants as a mean of supported rate of the variants. The numbers of examined and supported variants and the variant accuracies were presented in columns "Variants", "Supports", and "Accuracy" of Supplementary Table 7.

Supplementary References

1. Camacho C, Coulouris G, Avagyan V, Ma N, Papadopoulos J, Bealer K*, et al*. BLAST+: architecture and applications. *BMC Bioinformatics* 2009; **10:** 421.

2. Sievers F, Higgins DG. Clustal omega. *Curr Protoc Bioinformatics* 2014; **48:** 3 13 11-16.

3. Chin CS, Alexander DH, Marks P, Klammer AA, Drake J, Heiner C*, et al*. Nonhybrid, finished microbial genome assemblies from long-read SMRT sequencing data. *Nat Methods* 2013; **10**(6)**:** 563-569.

4. Robinson J, Halliwell JA, Hayhurst JD, Flicek P, Parham P, Marsh SG. The IPD and IMGT/HLA database: allele variant databases. *Nucleic Acids Res* 2015; **43**(Database issue)**:** D423-431.

5. Li H (2013). Aligning sequence reads, clone sequences and assembly contigs with BWA-MEM. In: *ArXiv e-prints*.

6. Aken BL, Ayling S, Barrell D, Clarke L, Curwen V, Fairley S*, et al*. The Ensembl gene annotation system. *Database* 2016; **2016:** baw093.

7. Katoh K, Misawa K, Kuma K, Miyata T. MAFFT: a novel method for rapid multiple sequence alignment based on fast Fourier transform. *Nucleic Acids Res* 2002; **30**(14)**:** 3059-3066.

8. Katoh K, Standley DM. MAFFT multiple sequence alignment software version 7: improvements in performance and usability. *Mol Biol Evol* 2013; **30**(4)**:** 772-780.

9. Nariai N, Kojima K, Saito S, Mimori T, Sato Y, Kawai Y*, et al*. HLA-VBSeq: accurate HLA typing at full resolution from whole-genome sequencing data. *BMC Genomics* 2015; **16 Suppl 2:** S7.

10. Nagasaki M, Yasuda J, Katsuoka F, Nariai N, Kojima K, Kawai Y*, et al*. Rare variant discovery by deep whole-genome sequencing of 1,070 Japanese individuals. *Nat Commun* 2015; **6:** 8018.

11. Handsaker RE, Van Doren V, Berman JR, Genovese G, Kashin S, Boettger LM*, et al*. Large multiallelic copy number variations in humans. *Nat Genet* 2015; **47**(3)**:** 296-303.

12. Rice P, Longden I, Bleasby A. EMBOSS: the European Molecular Biology Open Software Suite. *Trends Genet* 2000; **16**(6)**:** 276-277.

Supplementary Figures

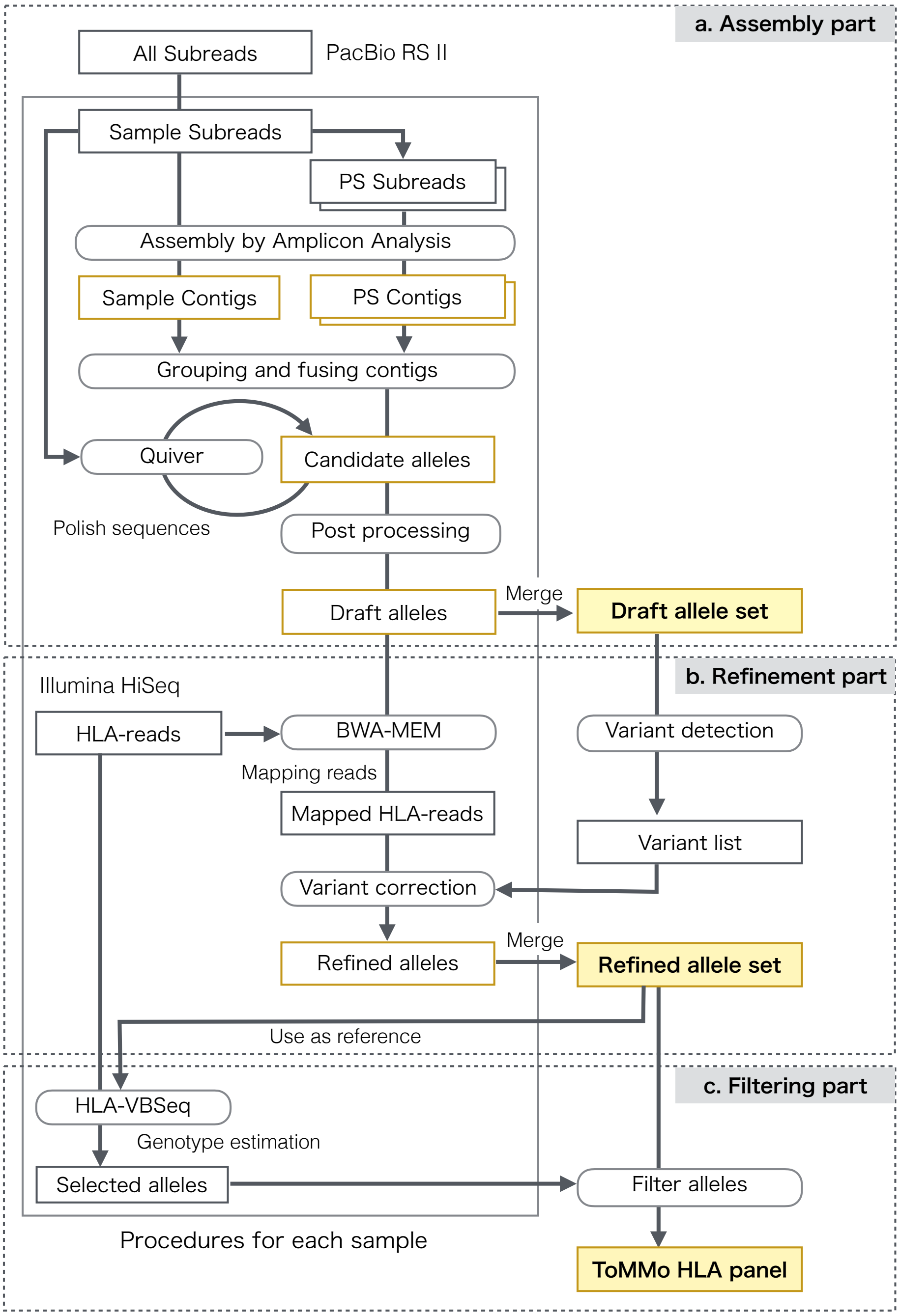

Supplementary Figure 1 | Flowchart of HLA panel construction with PSARP. Details of each part of PSARP are shown. (a) Assembly, (b) Refinement, and (c) Filtering.

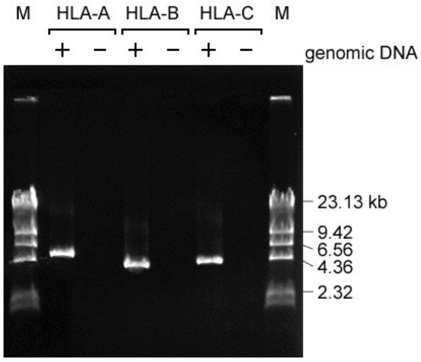

Lane	Sample
M1	Marker λ-Hind III digest
L1	positive control HLA-A_F/R
L2	negative control HLA-A_F/R
L3	positive control HLA-B_F/R
L4	negative control HLA-B_F/R
L5	positive control HLA-C_F/R
L6	negative control HLA-C_F/R
M2	Marker λ-Hind III digest

Supplementary Figure 2 | Electropherogram of PCR products. An electropherogram of PCR products for designed primer sets of HLA-A, -B, and -C genes is shown. The PCR product for each primer set was only observed in positive control sample

.

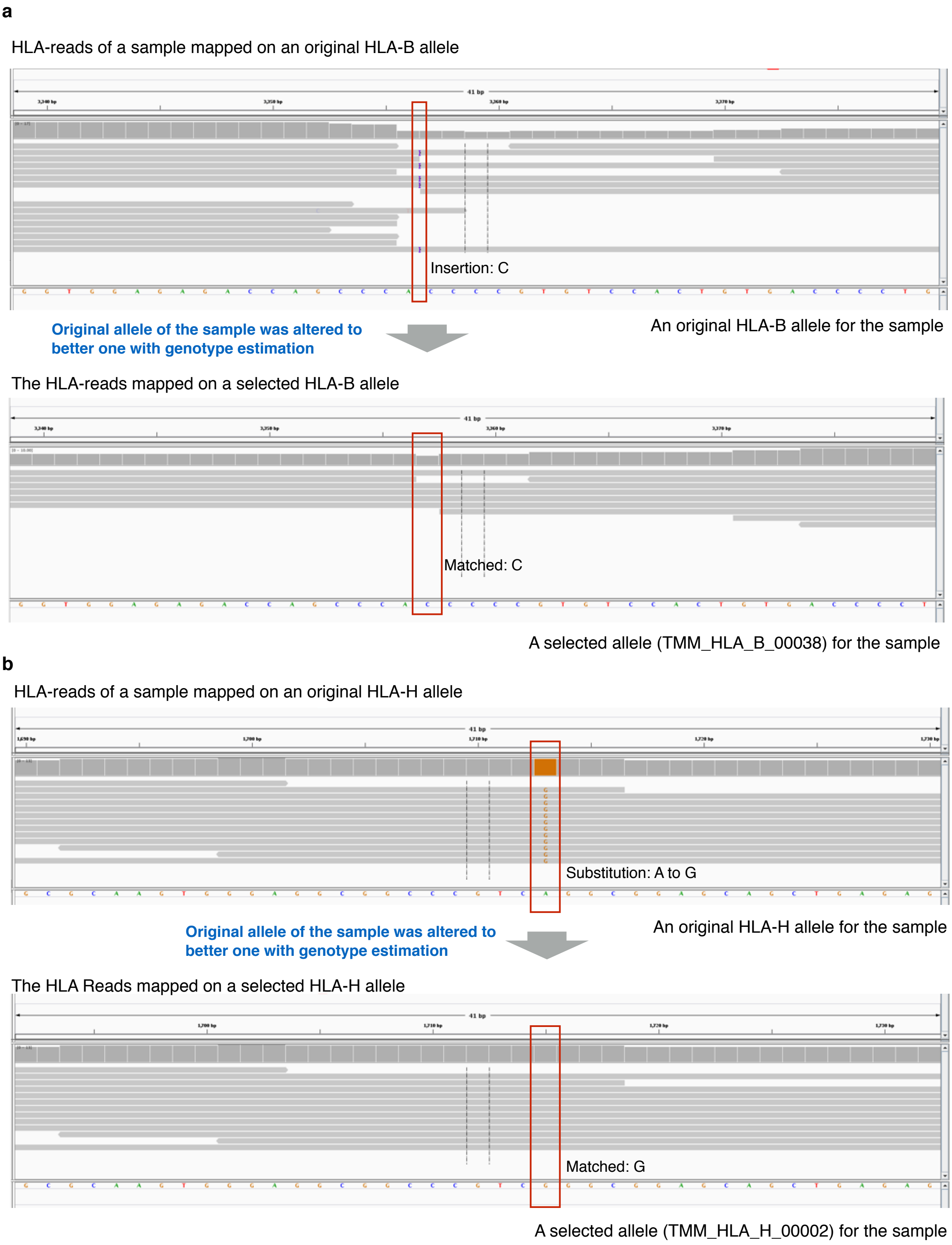

Supplementary Figure 3 | Examples of removed and selected alleles with the Filtering part of PSARP. Two examples of removed and selected alleles in the Filtering part of PSARP are shown. (a) HLA-reads of a sample, mapped on a removed HLA-B allele and the selected allele (TMM_HLA_B_00038) with genotype estimation are compared around position 3357 of the alleles. An insertion base ‘C’ present in the former allele is concordant with the selected allele. (b) HLA-reads of a sample, mapped on a removed HLA-H allele and the selected allele (TMM_HLA_H_00002) with genotype estimation are compared around positions 1713 of the alleles. A substitution of base ‘A’ to ‘G’ present in the former allele is concordant with the selected allele.

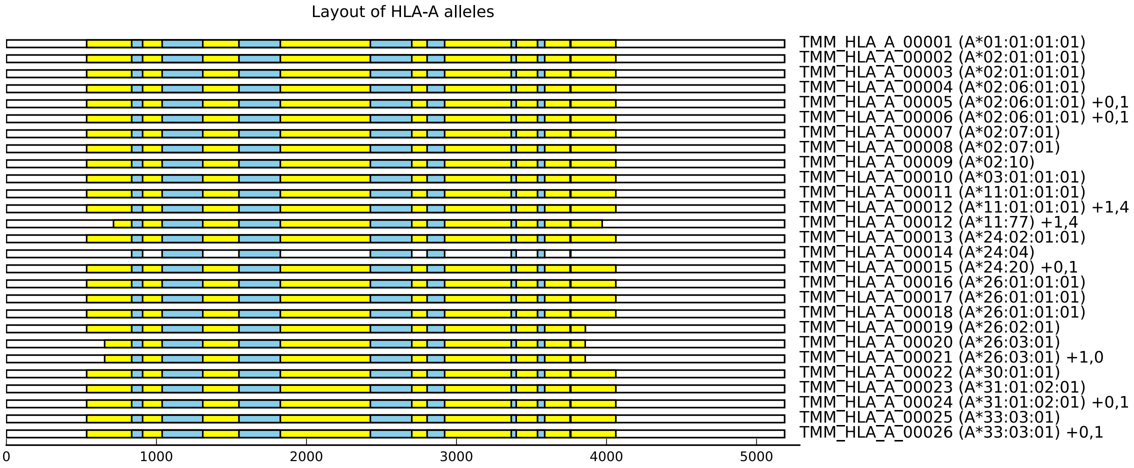

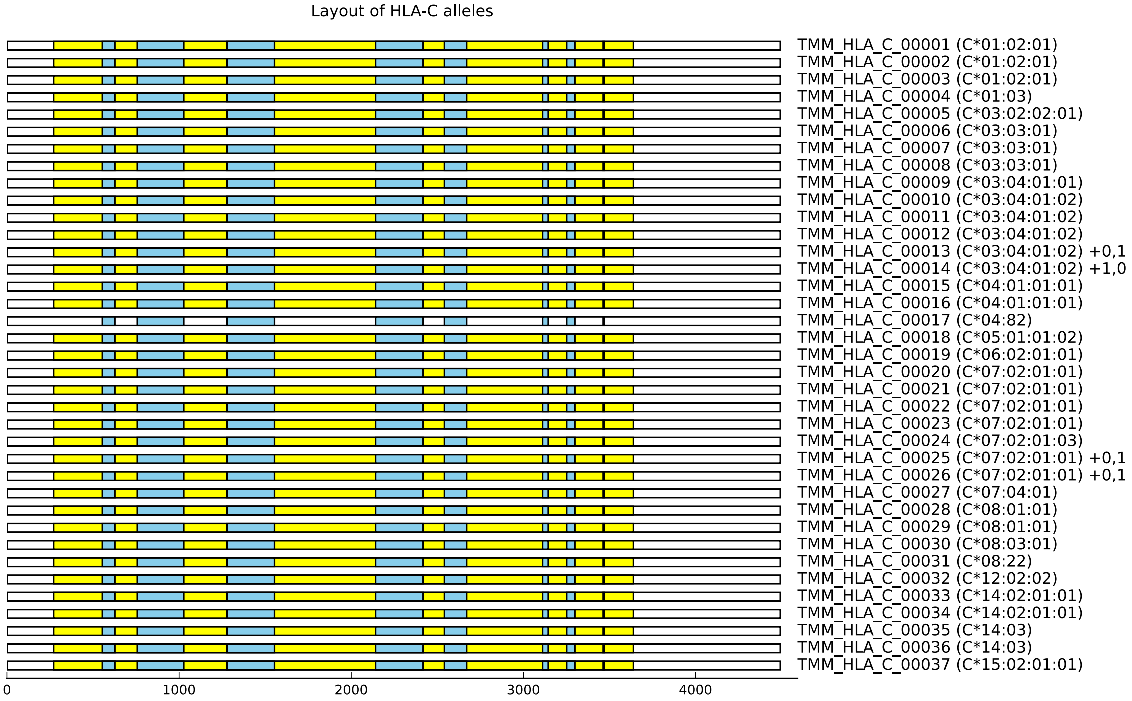

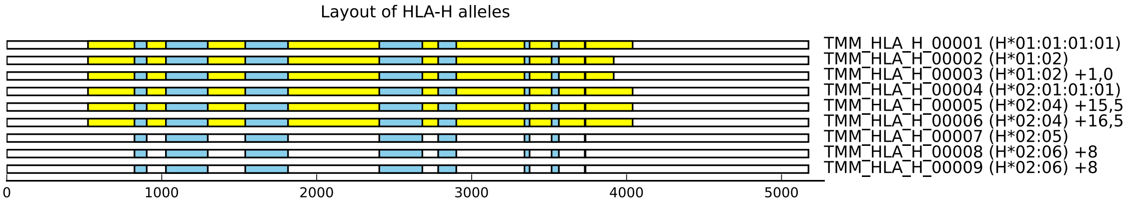

Supplementary Figure 4 | Layout of HLA-A, -C, and -H alleles. For each of HLA-A, -C, and -H genes, a layout of ToMMo HLA alleles and their closest subtypes in the IPD-IMGT/HLA database is shown. Blue, yellow, and white areas indicate exons registered in the database, the other genomic sequences in the database, and newly identified sequences in the panel, respectively. Each allele name is labelled in the right side of the layout. The closest subtype in the database is inside of the subsequent parentheses. The rightmost figures indicate edit distances between the allele and the closest subtype in exon region and those in the other region.

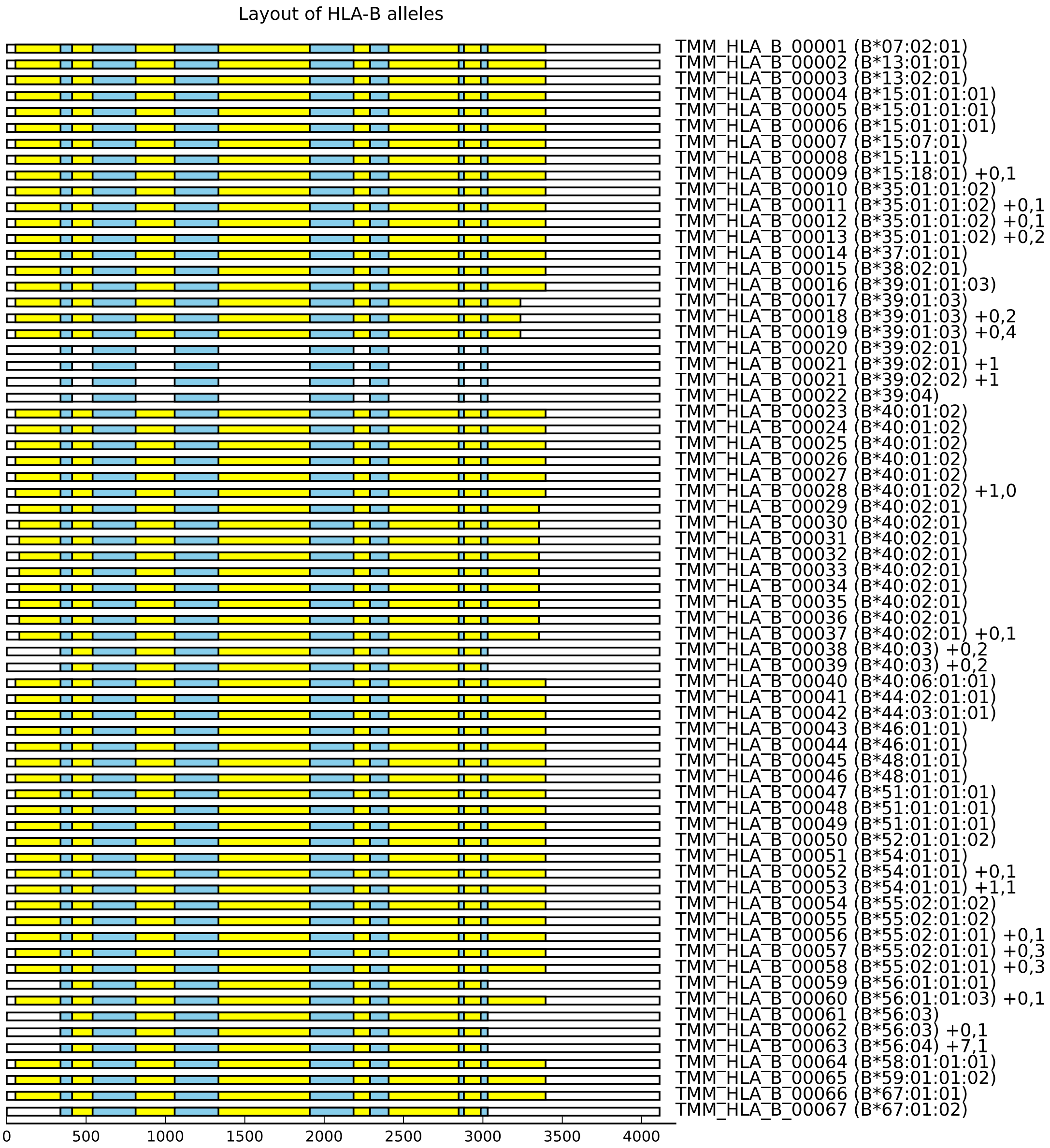

Supplementary Figure 5 | Layout of HLA-B alleles. A layout of ToMMo HLA alleles and their closest subtypes in the IPD-IMGT/HLA database is shown for the HLA-B gene. Notations are the same as those in Supplementary Figure 4.

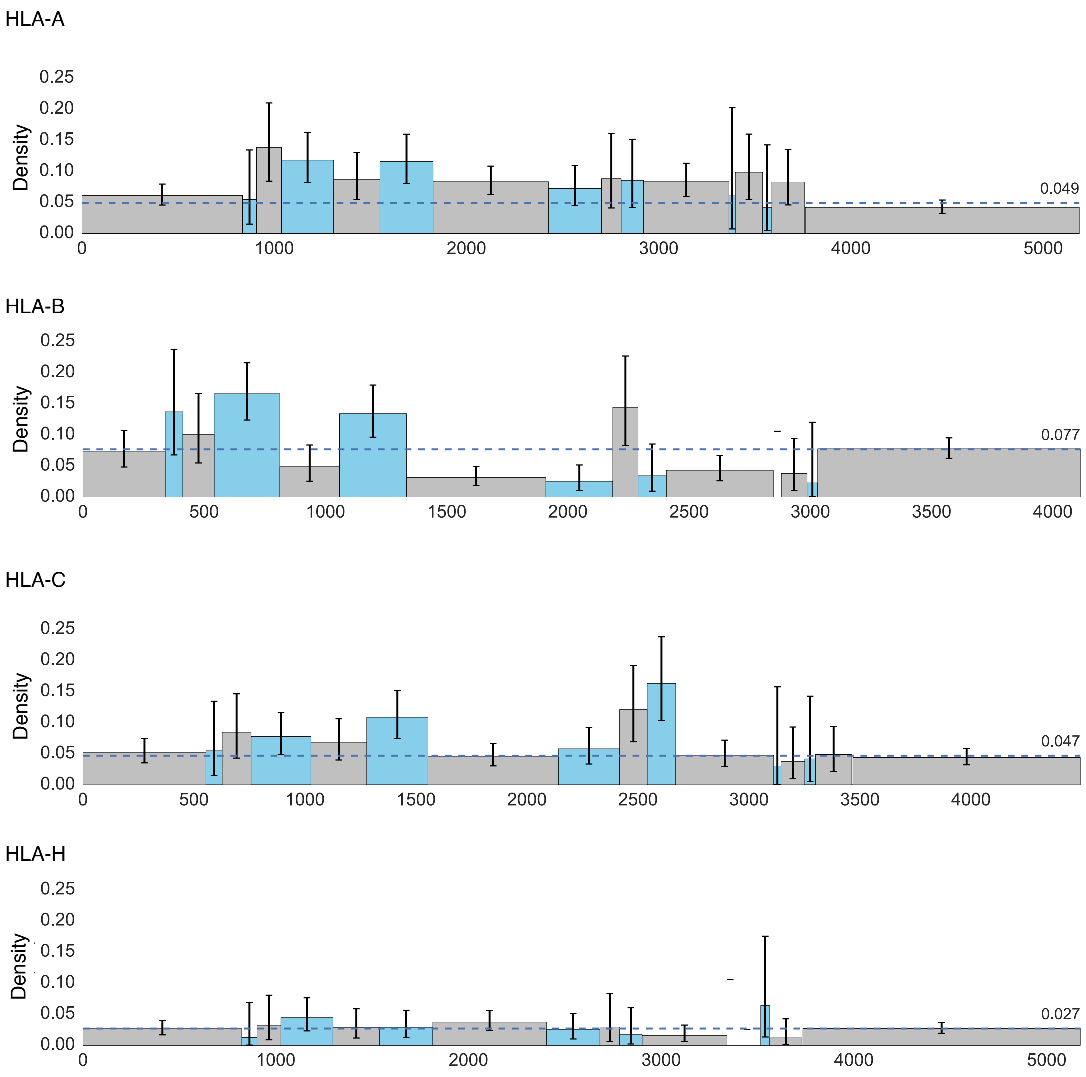

Supplementary Figure 6 | Density of variants in HLA-A, -B, -C, and -H alleles in the ToMMo HLA panel. Shown are density of variants and 95% confidence intervals in regulatory regions, exons, and introns for each of HLA-A, -B, -C, and -H alleles in the ToMMo HLA panel. Bars filled with sky blue colour correspond to exons. The dashed line is the mean density of variants for 3' upstream and 5' downstream regulatory regions.

Supplementary Tables

Supplementary Table 1 | PCR Primer locations and sequences

Locus	Coordinate on hg19		Forward Primer		Reverse Primer	
	Target region	Strand	Name	Sequence 5′ to 3′	Name	Sequence 5′ to 3′
HLA-A	chr6:29,909,497-29,914,680	+	A_F	GGACTCACACAGAAACTCAGAGC	A_R	AGGGTTCCTAAAGCATTCACTCC
HLA-B	chr6:31,321,157-31,325,297	-	B_F	CCCTGGTTTCCACAGACAGATCC	B_R	CACACTGCAGCACACAATCAGG
HLA-C	chr6:31,235,899-31,240,423	-	C_F	AGCTCACTGTCTGGCATCAAGTTCC	C_R	CTCAGGCCAAGTGCTGTTTTGTGG

Abbreviations: HLA, human leukocyte antigen.

Supplementary Table 4 | The number of samples for assembled contig numbers from initial analysis

HLA type	Number of assembled contigs								
	0	1	2	3	4	5	6	7	8
A	0	1	30	12	164	1	0	0	0
B	0	0	3	2	171	23	8	1	0
C	0	0	7	2	96	53	33	15	2
H	48	5	134	3	18	0	0	0	0

Abbreviations: HLA, human leukocyte antigen

Supplementary Table 5 | The number of full-length contigs from initial analysis

HLA type	Designed primer set	Assembled contigs	Full length contigs (rate)
A	A	758	690 (0.91)
B	B	866	853 (0.98)
C	C	986	289 (0.29)
H	(A)	354	326 (0.92)

Abbreviations: HLA, human leukocyte antigen

Supplementary Table 6 | The number of samples for full-length contig numbers from PSARP

HLA type	Number of full-length contigs		
	0	1	2
A	0	46	162
B	0	10	198
C	0	22	186
H	47	157	4

Abbreviations: HLA, human leukocyte antigen; PSARP, Primer-Separation Assembly and Refinement Pipeline

Supplementary Table 7 | The number of alleles and accuracies of variants in three allele sets

Allele set	HLA type	Alleles	Variant sites	Variants	Supports	Accuracy
Draft allele set	A	49	306	14,994	14,845	0.990
	B	167	223	37,241	35,784	0.961
	C	59	231	13,629	13,490	0.990
	H	61	131	7,991	7,759	0.971
	Total	336	891	73,855	71,878	0.973
Refined allele set	A	32	303	9,696	9,668	0.997
	B	115	221	25,415	24,570	0.967
	C	53	226	11,978	11,885	0.992
	H	48	123	5,904	5,825	0.987
	Total	248	873	52,993	51,948	0.980
ToMMo HLA panel	A	26	303	7,878	7,865	0.998
	B	67	221	14,807	14,582	0.985
	C	37	226	8,362	8,324	0.995
	H	9	123	1,107	1,105	0.998
	Total	139	873	32,154	31,876	0.991

Abbreviations: HLA, human leukocyte antigen; ToMMo, Tohoku Medical Megabank Organization.
